# Angiotensin-Converting Enzyme (ACE) Gene Insertion/Deletion Polymorphism and ACE Inhibitor-Related Cough: A Meta-Analysis

**DOI:** 10.1371/journal.pone.0037396

**Published:** 2012-06-19

**Authors:** Ya-Feng Li, Xiao-Ming Zhu, Fan Liu, Chuan-Shi Xiao, Yun-Fei Bian, Hong Li, Jun Cai, Rong-Shan Li, Xin-Chun Yang

**Affiliations:** 1 Department of Nephrology and Hemodialysis Center, Second Hospital of Shanxi Medical University, Taiyuan, Shanxi, China; 2 Department of Cardiology, Chaoyang Hospital, Capital Medical University, Beijing, China; 3 Department of Cardiology, Second Hospital of Shanxi Medical University, Taiyuan, Shanxi, China; 4 Medical College of Xiamen University, Xiamen, Fujian, China; Sapienza University of Rome, Italy

## Abstract

**Objective:**

An insertion/deletion (I/D) variant in the angiotensin-converting enzyme (ACE) gene was associated with ACE inhibitor (ACEI)–related cough in previous studies. However, the results were inconsistent. Our objective was to assess the relationship between the ACE I/D polymorphism and ACEI-related cough by meta-analysis and to summarize all studies that are related to ACE I/D polymorphism and ACEI-cough and make a summary conclusion to provide reference for the researchers who attempt to conduct such a study.

**Methods:**

Databases including PubMed, EMbase, Cochrane Library, and China National Knowledge Infrastructure, were searched for genetic association studies. Data were extracted by two independent authors and pooled odds ratio (OR) with 95% confidence interval (CI) was calculated. Metaregression and subgroup analyses were performed to identify the source of heterogeneity.

**Results:**

Eleven trials, including 906 cases (ACEI-related cough) and 1,175 controls, were reviewed in the present meta-analysis. The random effects pooled OR was 1.16 (95%CI: 0.78–1.74, *p* = 0.46) in the dominant model and 1.61 (95%CI: 1.18–2.20, *p* = 0.003) in the recessive model. Heterogeneity was found among and within studies. Metaregression indicated that the effect size was positively associated with age and negatively associated with follow-up duration of ACEI treatment. Subgroup analysis revealed a significant association between ACE I/D polymorphism and ACEI-related cough in studies with mean age >60 y, but not in studies with mean age ≤60 y. No heterogeneity was found within each mean age subgroup. We also found no association between ACE I/D polymorphism and ACEI-related cough in studies with follow-up>2 mo or in studies in Caucasians. No heterogeneity was detected in these two subgroups.

**Conclusions:**

Synthesis of the available evidence supports ACE I/D polymorphism as an age-dependent predictor for risk of ACEI-related cough.

## Introduction

Angiotensin-converting enzyme inhibitors (ACEI) have been used for 30 years to treat cardiovascular diseases. In addition to their antihypertensive effects, ACEI are now widely used for congestive heart failure, acute myocardial infarction, and diabetic nephropathy [1]–[5]. However, a persistent dry cough associated with ACEI therapy is the most common adverse effect and the most frequent reason for discontinuation of the drug [6], [7]. The prevalence of cough varies from 0.7% to 53% worldwide and has been reported to be higher in Asian populations than in Caucasian populations [8]–[12].

The pathogenesis of ACEI-related cough remains unclear. It has been speculated that a local accumulation of bradykinin may play a major role in ACEI-related cough. This accumulation may lead to the activation of proinflammatory peptides (e.g., substance P, prostaglandins, neuropeptide Y, and phospholipase C and/or A2) and local release of histamine in the airways [6], [7]. ACE plays an essential role as a kininase in the degradation of bradykinin, and has a higher affinity for bradykinin than for angiotensin I [13], [14].

An insertion/deletion (ID) polymorphism of the human ACE gene on chromosome 17q23 comprising the presence or absence of a 287 base pair (bp) insert in intron 16 was described by Rigat et al. in 1990 [15]. Subsequently, some studies confirmed that the ID polymorphism is in strong linkage disequilibrium with a major gene effect at the ACE gene locus, which controls up to 16–24% of the variability in ACE levels [16], [17]. Patients with genotype II have the lowest serum ACE levels compared to those with ID or DD [16]. Therefore, genotype II would be expected to be associated with an increased risk of ACEI-related cough. Although the results of several studies supported this hypothesis [18], [19], but the relationship was not replicated in other studies [20]–[22]. In the present study, we used a meta-analysis to address these discrepancies and to investigate the association between the ID polymorphism and ACEI-related cough.

## Methods

### Database Searches

Using the term “ACE” or “ACEI” paired with “cough” and “polymorphism” without language restriction, we systematically searched PubMed (http://www.ncbi.nlm.nih.gov/pubmed; 1950–March 2011), EMbase (http://www.embase.com; 1950–March 2011), China National Knowledge Infrastructure (CNKI) (http://www.cnki.net; 1979–March 2011), the Cochrane Library (http://www.cochrane.org), and reviews and reference lists of relevant articles. The last search was performed in March 2011.

### Study Selection

We selected studies as follows: (1) unrelated case-control studies and cohort studies; (2) complete data with genotype and allele frequencies; (3) genotype frequency of cases and controls is within Hardy–Weinberg equilibrium; and (4) studies in which the ID polymorphism has been determined. All of the literature searches were independently reviewed by two of the authors (Ya-Feng Li and Yun-Fei Bian).

### Data Extraction

We extracted the following information from each study: first author, journal, year of publication, study population, demographics, number of cases and controls for ID genotype, type and dosage of ACEI, and follow-up time. The frequencies of the alleles and the genotypic distributions were extracted or calculated for both the cases and the controls.

### Statistical Analysis

Data from the meta-analysis were analyzed using Stata software (Version 9.0; Stata Corportation, College Station, TX, USA) and REVMAN software (Version 5.0; Cochrane Collaboration, Oxford, UK). The significance of the association for the dominant model (II/ID versus DD genotypes), the recessive model (II versus ID/DD genotypes), and the allele contrast I versus D were evaluated for 11 studies separately. All associations were indicated as odds ratios (ORs) with 95% confidence interval (CI). The pooled ORs were estimated using random effects (RE) and fixed effects (FE) models based on the individual Ors [23].

Statistical heterogeneity between studies was formally tested with Cochran’s test. We also determined the *I^2^* statistic, which yields values between 0% and 100%, with higher values denoting greater degree of heterogeneity (0%≤*I^2^*<25%: no heterogeneity; 25%≤*I^2^*<50%: moderate heterogeneity; 50%≤*I^2^*<75%: large heterogeneity; and 75%≤*I^2^*<100%: extreme heterogeneity) [24]. Metaregression was used to explore the source of potential heterogeneity. Subgroup analyses were performed to further identify the possible source of heterogeneity by comparing results obtained from subsets of studies grouped by mean age of cases, proportion of males, and study population.

Publication bias was assessed with the Egger regression test and represented graphically by use of Begg’s funnel plots of logOR compared with the standard error (SE) of logOR [25].

A chi-square (χ^2^) test was applied to determine if genotype distributions of the control population reported conformed to Hardy-Weinberg equilibrium (HWE; P<0.05 was considered significant), and the study that the genotype distributions in the controls were significantly deviated from HWE was excluded from our sensitive analysis [26]. All *p* values were two-tailed and *p*<0.05 was considered statistically significant.

## Results

### Search Results

A total of 18 case-control studies and cohort studies were identified in a systematic search of PubMed, EMbase, CNKI, Cochrane Library, and references cited in reviews and research articles. Six studies were excluded because they were not within Hardy–Weinberg equilibrium (Table 1) [27]–[32]. One study was excluded because the frequencies of the genotypic distributions cannot be extracted from the paper [33]. Eleven studies were finally included in this meta-analysis [18]–[22], [34]–[39]. Preferred Reporting Items for Systematic Reviews and Meta-Analyses (PRISMA) checklist and flow diagram were respectively stated in Table S1 and Figure S1.

**Table 1:** The Distribution of the I/D Genotype for Cases and Controls.

References	Year	Cases						Controls								
		Distribution of I/D genotype			Allele Frequency		HWE *P*	Distribution of I/D genotype			Allele Frequency				HWE *P*	
		II	ID	DD	I	D		II	ID	DD	I		D			
**W. W. Yeo, ** ***et al*** **.**	1992	5	6	7	16	20	**>0.05**	2	6	7	10	20		**>0.05**		
**K. Furuya, ** ***et al.***	1994	19	12	0	50	12	**>0.05**	25	35	11	85	57		**>0.05**		
**C. Kreft-Jais, ** ***et al.***	1994	15	29	27	59	83	**>0.05**	12	33	30	57	93		**>0.05**		
**I.G. Chadwick, ** ***et al.***	1994	6	12	13	24	38	**>0.05**	58	97	66	213	229		**>0.05**		
**R.Y. Zee, ** ***et al.***	1998	23	41	35	87	111	**>0.05**	12	37	21	61	79		**>0.05**		
Je Hyeong Kim, *et al.*	1999	6	7	24	19	55	<0.05	57	55	290	169	635		<0.05		
Wu Jianqing, *et al.*	1999	24	15	13	63	41	<0.05	21	19	30	61	79		<0.05		
**H. Okumura, ** ***et al.***	2000	20	16	6	56	28	**>0.05**	19	31	4	69	39		**>0.05**		
**Y-J Lee, ** ***et al.***	2001	46	44	3	136	50	**>0.05**	33	48	15	114	78		**>0.05**		
S Mukae, *et al.*	2002	8	45	17	61	79	<0.05	28	73	19	129	111		<0.05		
Wang huijuan, *et al.*	2003	18	14	8	50	30	**>0.05**	35	36	34	106	104		<0.05		
**S-M Yang, ** ***et al.***	2003	21	20	11	62	42	**>0.05**	10	21	19	41	59		**>0.05**		
**J Lu, ** ***et al.***	2003	148	163	40	459	243	**>0.05**	160	165	40	485	245		**>0.05**		
Ye Ruan-jia, *et al.*	2004	27	14	7	68	28	<0.05	16	35	28	67	91		**>0.05**		
Shi Meijun, *et al.*	2008	28	15	8	71	31	<0.05	17	36	28	70	92		**>0.05**		
**Tang Xiao-Hong, ** ***et al.***	2008	32	26	10	90	46	**>0.05**	24	48	26	96	100		**>0.05**		
**S. W. Woo, ** ***et al.***	2009	26	16	8	68	32	**>0.05**	24	31	5	79	41		**>0.05**		

References in bold-face were studies within Hardy–Weinberg equilibrium.

### Study Characteristics

In total, the study contained 906 hypertensives with ACEI-related cough as cases and 1,175 hypertensives without ACEI-related cough as controls. Table 2 shows the details of these studies. The sample size of the 11 studies ranged from 18 to 351 cases. The average age of ACEI-related cough subjects in the 11 studies ranged from 53 to 72.1 y (median age: 60 y). The percentage of males in the studies ranged from 18.3%–71.2% (median: 62%). The ACEIs used in the studies varied. The duration of follow-up of ACEI treatment varied from 0.5 to 36 mo (median: 2 mo). Five studies matched the case and control groups with regard to age and sex [34]–[37], [39]. Three studies were not age- or sex-matched [18], [19], [22], and we could not obtain this information for the remaining three studies [20], [21], [38]. The populations of seven studies were Asian [18], [19], [35]–[39], and the remaining four study populations were Caucasian [20]–[22], [34].

**Table 2:** Characteristics of Eligible Studies Considered in the Meta-Analysis.

Reference	Population	ACEIs	Dose (mg/d)	Interval (months)	Case			Control			Age- and sex- Matched
					Mean age (*y*)	Number	Male *%*(n)	Mean age (*y*)	Number	Male *%*(n)	
20	Caucasian	enalapril (81.3%)	20	6	53	71	–	47	75	–	Unknown
34	Asian	benazepril	–	36	55.4	351	52.7 (185)	–	365	56.2(205)	Yes
21	Caucasian	lisinopril	20	1.5	57	99	47.5(47)	58.4	70	34.3(24)	No
31	Caucasian	enalapril	10–20	6	–	18	–	–	15	–	Yes
36	Asian	–	–	3	58.5	50	62.0(31)	58.3	60	61.7(37)	Yes
32	Asian	trandolapril	1	1	60	42	64.3(27)	59	54	53.7(29)	Yes
35	Asian	captopril	50	2	61.8	68	–	–	98	–	Unknown
18	Asian	perindopril	4	0.5	63.3	93	18.3(17)	58.6	96	68.8(66)	No
17	Asian	enalapril (75.5%)	20	–	65	31	51.6(16)	58	71	50.7(36)	No
33	Asian	lisinopril	–	1	72.1	52	71.2(37)	72.6	50	78.0(39)	Yes
19	Caucasian	enalapril	–	–	–	31	–	–	221	–	Unknown

–, not reported.

### Association between Genotypes and ACEI-related Cough

Table 1 shows the genotype distribution and allele frequencies in the original 18 studies. In the 11 studies that were within Hardy–Weinberg equilibrium [18]–[22], [34]–[39], the distributions of genotypes II and DD were significantly different between Asian and Caucasian study populations (II: 36.4% versus 24.9%, *p* = 0.0001 and DD: 16.3% versus 28.6%, *p*<0.0001, respectively, in Asians and Caucasians). No difference was found in the distribution of genotype ID between Asians and Caucasians (47.3% versus 46.5%, respectively; *p* = 0.8430).

**Figure 1:**
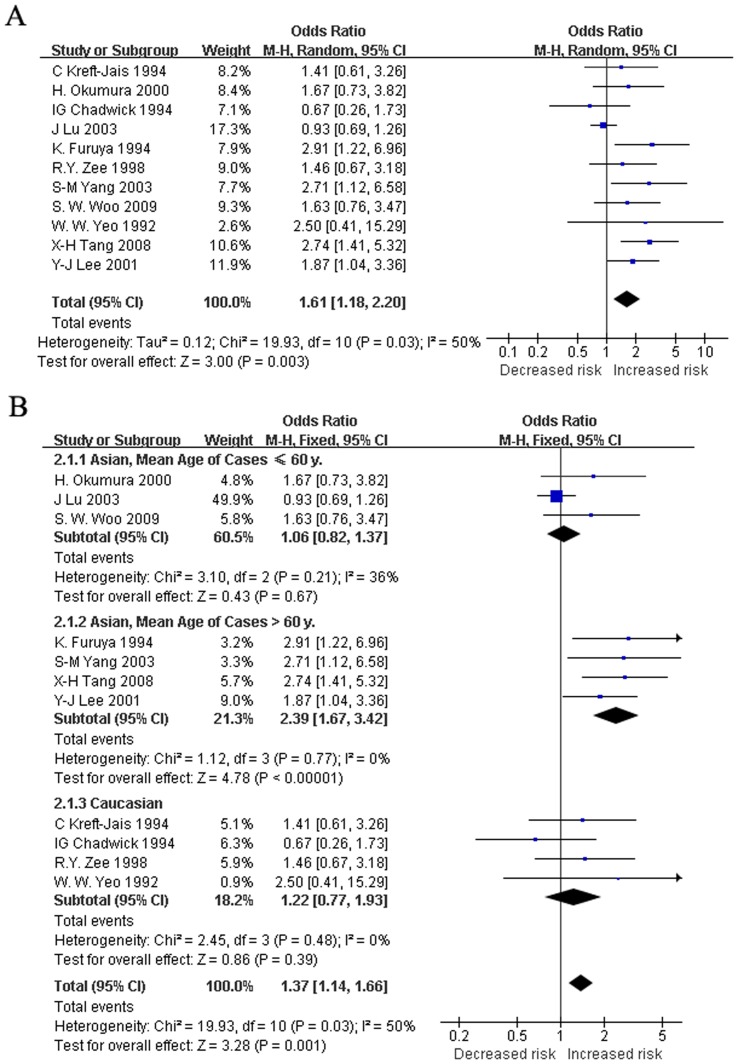
Random effect (A) and fixed effect (B) pooled ORs for the recessive model. Subgroup analyses in recessive model were also carried out (B). Studies were first divided by ethnicity, then Asian population studies were divided into two subgroups: ≤60 y old (lower median) and >60 y old (upper median). The fixed-effect model was used for the analysis in (B), because heterogeneity was reduced in subgroup analyses.

The RE and FE pooled ORs for the recessive model were 1.61 (95%CI: 1.18 to 2.20, *p* = 0.003) and 1.37 (95%CI: 1.14 to 1.66, *p* = 0.001), respectively (Figure 1A, 1B). However, we found heterogeneity of the pooled ORs (*I^2^* = 49.8%, *p* = 0.030). To determine the sources of heterogeneity, metaregression and subgroup analyses were performed. Metaregression analysis of data showed that the mean age of hypertensives with ACEI-related cough and the follow-up duration of ACEI treatment contributed to the heterogeneity. The mean age was positively associated with the OR (regression coefficient = 0.11, 95%CI: 0.05 to 0.17, *p* = 0.001), and the duration of treatment was negatively associated with the OR (regression coefficient = −0.03, 95%CI: −0.05 to −0.01, *p* = 0.008). The type of ACEI, the proportion of males, and the study population were not effect modifiers.

To further investigate the heterogeneity, we performed subgroup analyses (Table 3). Other than mean age, duration of treatment, and study populations, we did not find any evidence of heterogeneity of effects in the subgroup analyses, including the category of ACEI or the sex ratio. We calculated the median value of the mean ages of hypertensives with ACEI-related cough and defined the mean age ≤60 y (lower median) for all studies to be one subgroup; the mean age >60 y (upper median) was assigned to the other subgroup. No heterogeneity existed in either the younger median subgroup (≤60 y) or the older median subgroup (>60 y) (*I* ^2^ = 1%, *p* = 0.403 and *I* ^2^ = 0%, *p* = 0.773; respectively). The FE pooled ORs (1.12 [95%CI: 0.88 to 1.42, *p* = 0.360] versus 2.39 [95%CI: 1.67 to 3.42, *p*<0.001]) were significantly different between the two mean age subgroups (*p*<0.05). In addition, when subgroups were categorized by the median of follow-up duration of ACEI treatment, heterogeneity was also reduced in both the shorter duration median subgroup (≤2 mo) and the longer duration median subgroup (>2 mo) (*I* ^2^ = 0%, *p* = 0.705 and *I* ^2^ = 7%, *p* = 0.359; respectively). The FE pooled ORs of the two ACEI treatment duration median subgroups different significantly (2.02 [95%CI: 1.47 to 2.79, *p*<0.001] versus 1.06 [95% CI: 0.82, 1.37, *p* = 0.660]) (*p* = 0.0016).

**Table 3:** Subgroup analyses for dominant model, recessive model and the allele contrast I versus D.

	Intervention group (n)	Dominant model			Recessive model			The allele contrast I versus D		
		*I^2^*	RE pooled ORs (95% CI)	*P*	*I^2^*	RE pooled ORs(95% CI)	*P*	*I^2^*	RE pooled OR (95% CI)	*P*
Mean age										
≤60 y, low median	5	0%	0.87 (0.64, 1.18)	**<0.05**	1%	1.12 (0.88, 1.42)	**<0.05**	0%	1.01 (0.85, 1.20)	**0.0001**
>60 y, high median	4	0%	2.71 (1.60, 4.61)		0%	2.39 (1.67, 3.43)		0%	2.07 (1.61, 2.68)	
Category of ACEIs										
enalapril (or major)	4	42%	1.04 (0.52, 2.09)	0.4307	44%	1.52 (0.77, 3.01)	0.6334	70%	1.30 (0.73, 2.35)	0.7469
others	7	63%	1.23 (0.73, 2.09)		58%	1.65 (1.14, 2.40)		67%	1.36 (1.02, 1.81)	
Interval of treatment										
>2 months	4	0%	0.96 (0.67, 1.37)	0.1155	7%	1.09 (0.81, 1.47)	**0.0016**	0%	0.95 (0.79, 1.14)	**0.0023**
≤2months	5	67%	1.56 (0.75, 3.24)		0%	2.03 (1.47, 2.80)		50%	1.57 (1.15, 2.13)	
Proportion of male										
<60%, low median	4	72%	1.59 (0.67, 3.80)	0.2530	0%	1.85 (1.29, 2.66)	0.2895	78%	1.39 (0.90, 2.17)	1.0000
>60%, high median	3	67%	0.87 (0.28, 2.69)		67%	1.46 (0.75, 2.83)		40%	1.39 (0.91, 2.13)	
Population										
Asian	7	65%	1.49 (0.78, 1.74)	**0.0012**	65%	1.82 (1.20, 2.74)	**0.0298**	73%	1.55 (1.11, 2.16)	**0.0042**
Caucasian	4	0%	0.85 (0.58, 1.25)		0%	1.23 (0.77, 1.98)		5%	0.99 (0.76, 1.31)	

We also found reduced heterogeneity (*I^2^* = 0%; *p* = 0.485) and an FE pooled OR of 1.22 (95%CI: 0.77 to 1.93, *p* = 0.390) for the Caucasian studies. However, the seven Asian population studies were highly heterogeneous (*I^2^* = 65.2%, *p* = 0.008) (Table 3). Therefore, we divided the seven Asian studies into two subgroups according to the mean age of hypertensives with AECI-related cough: ≤60 y (lower median) and >60 y (upper median). The heterogeneity was reduced in each of the subgroups (*I^2^* = 36%, *p* = 0.212; *I^2^* = 0%, *p* = 0.773; respectively), and the FE pooled ORs were 1.06 (95%CI: 0.82 to 1.37, *p* = 0.668) and 2.39 (95%CI: 1.67 to 3.42, *p*<0.001) (Figure 1B).

**Figure 2:**
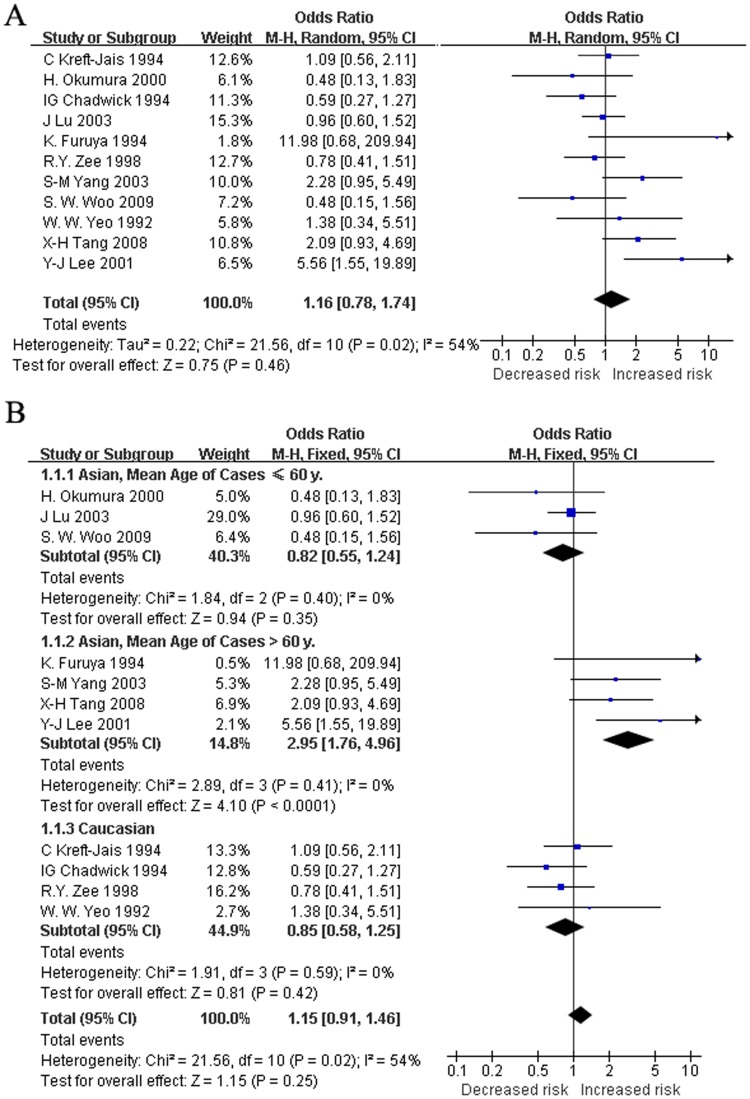
Random effect (A) and fix effect (B) pooled ORs for the dominant model. Subgroup analyses were also carried out (B). In each subgroup, heterogeneity was reduced.

We also compared the II/ID genotypes to the DD genotype with the extracted data pooled from the 11 studies. The RE and FE pooled ORs for the dominant model were 1.16 (95%CI: 0.78 to 1.74, *p* = 0.46) and 1.15 (95%CI: 0.91 to 1.46, *p* = 0.25), respectively, and a moderate heterogeneity was found (*I^2^* = 54%, *p* = 0.02) (Figure 2A, 2B). We did not find heterogeneity when subgroups were categorized by mean age (Table 3, data not shown). The follow-up duration of ACEI treatment and the study population reduced the heterogeneity in one set of subgroups (for follow-up >2 mo: *I* ^2^ = 0%, for Caucasians: *I* ^2^ = 0%), but the heterogeneity in the other subgroup was still large (for interval ≤2 mo: *I* ^2^ = 67%, for Asians: *I* ^2^ = 65%) (Table 3). For the dominant model, the FE OR was 0.95 (95%CI: 0.67 to 1.35, *p* = 0.787) in studies with a >2 mo follow-up duration of ACEI treatment, and the FE OR was 0.85 (95%CI: 0.58 to 1.25, *p* = 0.591) in the Caucasian study population. Further, we divided the 11 studies into three subgroups: Asians with mean age of cases ≤60 y, Asians with mean age of cases >60 y, and Caucasians. In each subgroup, heterogeneity was reduced (*I^2^* = 0%, *p* = 0.399; *I^2^* = 0%, *p* = 0.409; *I^2^* = 0%, *p* = 0.591; respectively). The FE pooled ORs were 0.82 (95%CI: 0.55 to 1.24, *p* = 0.348), 2.95 (95%CI: 1.76 to 4.96, *p*<0.001), and 0.85 (95%CI: 0.58 to 1.25, *p* = 0.419), respectively (Figure 2B).

**Figure 3:**
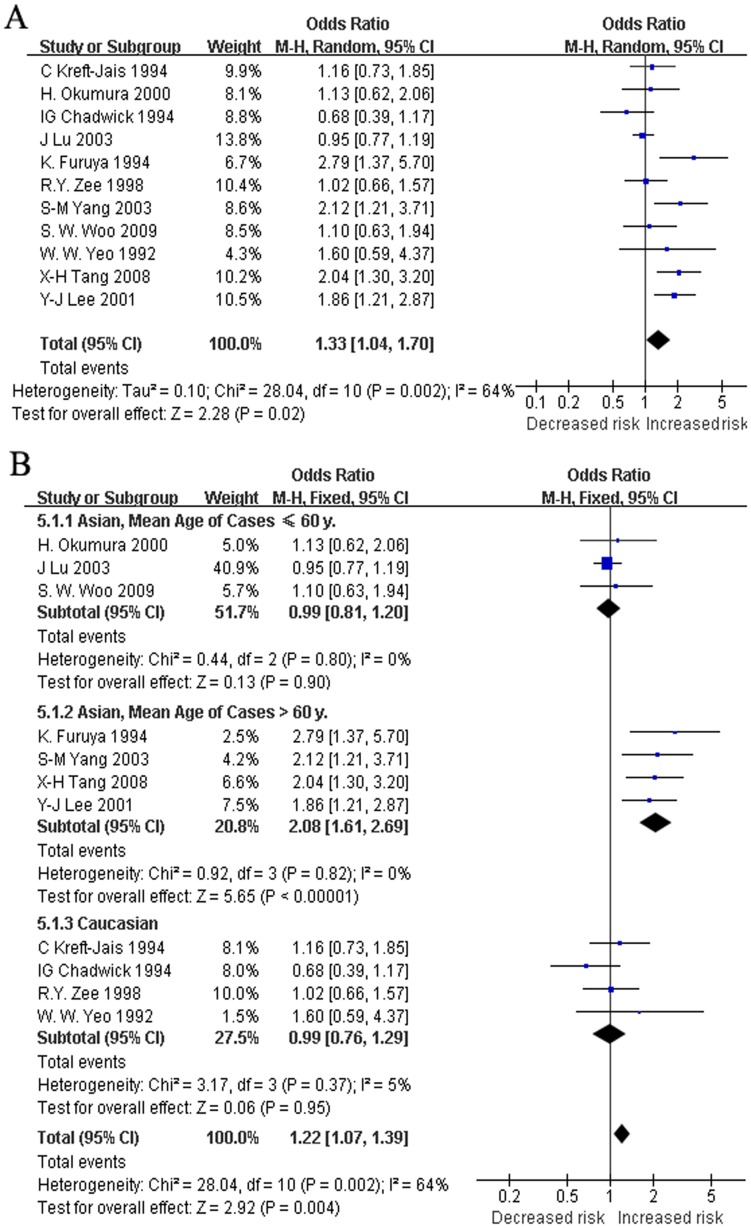
Random effect (A) and fix effect (B) pooled ORs for the contrast between alleles I and D. Subgroup analyses were also carried out (B). In each subgroup, heterogeneity was reduced. Allele I was a predictor of ACEI-related cough only in the subgroup of Asians >60 y old.

There was high heterogeneity when allele I was contrasted with allele D (*I^2^* = 64%, *p* = 0.002), and the RE and FE pooled ORs were 1.33 (95%CI: 1.04 to1.70, *p* = 0.02) and 1.22 (95%CI: 1.07 to 1.39, *p* = 0.004) (Figure 3A, 3B). No heterogeneity existed within each of the mean age subgroups. Heterogeneity did exist among Asian studies and studies with a follow-up duration of ≤2 mo, but not among Caucasian studies or studies with a follow-up duration of >2 mo (Table 3). The FE pooled OR was 0.95 (95%CI: 0.79 to 1.14, *p* = 0.572) in studies with a follow-up duration of >2 mo and 0.99 (95%CI: 0.76 to 1.29, *p* = 0.952) in the Caucasian studies. We further divided the studies by population and mean ages. Among the three subgroups (Asians with mean age of cases ≤60 y, Asians with mean age of cases >60 y, and Caucasians), no heterogeneity was detected (*I^2^* = 0%, *p* = 0.80; *I^2^* = 0%, *p* = 0.82; *I^2^* = 5%, *p* = 0.37; respectively), and the FE pooled ORs were 0.99 (95%CI: 0.81 to 1.20, *p* = 0.90), 2.08 (95%CI: 1.61 to 2.69, *p*<0.00001), and 0.99 (95%CI: 0.76 to 1.29, *p* = 0.95), respectively (Figure 3B).

### Publication Bias

**Figure 4:**
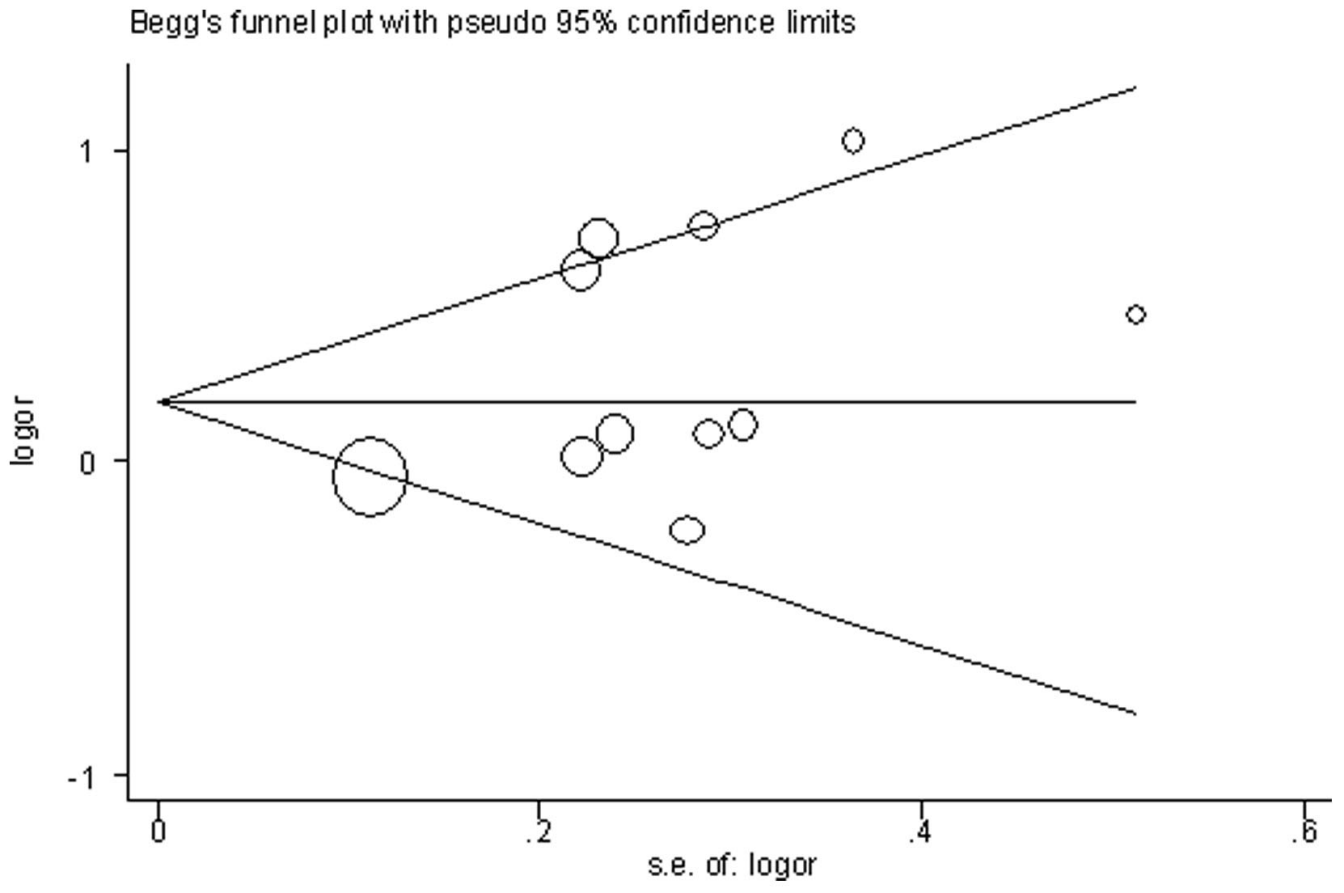
Funnel plot of SE against the log OR for I/D polymorphism.

A statistical analysis of the Egger test and funnel plots was performed for all 11 studies (Figure 4). No publication bias was detected for the contrast of allele I versus D (Egger test, *p* = 0.102).

## Discussion

Our meta-analysis of 11 studies revealed that a greater I allele frequency gave rise to an increased susceptibility to ACEI-related cough. However, heterogeneity was detected in the 11 studies. The meta-regression analysis indicated that the effect size was positively related to the mean age of the subjects and negatively related to the follow-up duration.

In the subgroup analysis, the association depended on three variables: (1) Age: a strong association between the ACE I/D polymorphism and ACEI-related cough was only identified in studies where the mean age was >60 y, not in studies where the mean age was ≤60 y. (2) Follow-up duration: no significant association existed between the ACE I/D polymorphism and ACEI-related cough when the follow-up duration was >2 months. (3) Ethnicity: we did not find any association between the ACE I/D polymorphism and ACEI-related cough in studies on Caucasian populations. This may have resulted from differences in age among studies, because the mean age of patients in two of the Caucasian groups was <57 y [21], [22]; in the other two Caucasian studies, the age could not be extracted [20], [34].

The mechanism for ACEI-related cough was not entirely clear. We believe that the lower level of tissue ACE (which correlates with a lower level of blood ACE in the patient) leads to higher concentrations of ACE substrates (both bradykinin and substance P), which may contribute to cough development. Bradykinin, a peptide degraded by ACE, has been suggested to play a major role in ACEI-related cough [13], [14]. The accumulation of bradykinin may induce activation of proinflammatory peptides (e.g., substance P), increase the sensitization of airway sensory nerves, and increase coughing [6], [7], [40]–[42]. Higher levels of substance P have been found in patients with ACEI-related cough [43]. The I/D polymorphism of ACE was previously found to be associated with serum ACE activity. Subjects with genotypes II, ID, and DD, showed low, intermediate, and high ACE activity, respectively [15], [16]. This supported the notion that the ACE I/D polymorphism was associated with ACEI-related cough. However, many other studies did not find an association between the ACE I/D polymorphism and ACEI-related cough [20]–[22], [37].

Some studies indicated that the ACE I/D polymorphism was associated with cough sensitivity, rather than ACEI-related cough (cough sensitivity was defined as a cough reflex response to stimulation with distilled water) [20], [44]. Morice *et al.* speculated that ACEI induced a slow, marked reduction in ACE activity, but the loss of activity might be compensated by other peptide-degrading pathways. For example, neutral endopeptidase (NEP), which has been shown to regulate substance P and cough, was one of the candidates for these compensating pathways [45]. Another study investigated the association between the ACE gene polymorphism and the cough threshold (the number of coughs in response to capsaicin over a 5-min interval) after cilazapril (an ACEI) administration; those results supported the notion of compensation. In that study, serum ACE activities were effectively inhibited by the ACEI, and ACE activities in the subjects with genotype II showed significant differences from those with genotype DD. However, no significant differences in plasma bradykinin or substance P were found, either before or after the administration of ACEI in subjects with genotype II or DD [46]. The mean age of the subjects in the study was <20 y. In youths, bradykinin can be sufficiently degraded by peptide-degrading pathways. The degradation of bradykinin by a variety of peptidases, including dipeptidyl-aminopeptidase IV (DPIV), aminopeptidase N (APN), or neutral aminopeptidase, may be substantially enhanced in an environment where ACEI has reduced ACE activity [47].

Based on dominant and recessive gene models, we estimated the effects of an ACE gene polymorphism on ACEI-related cough. We found that the I allele of the ACE gene increased the susceptibility to ACEI-related cough. However, due to heterogeneity among the 11 studies, we performed a meta-regression analysis. This indicated that the effect size was positively related to the mean age of the subjects and negatively associated with the follow-up duration after ACEI treatment. The meta-analysis results also supported the notion that an ACEI-induced reduction in ACE activity may lead to bradykinin accumulation, which may contribute to ACEI-related cough. However, when the ACEI treatment is long in duration, bradykinin could be degraded by alternative metabolic pathways, and the ACEI-related cough might eventually cease or become tolerable. In some long-term studies, subjects that had recovered from an ACEI-related cough due to delayed bradykinin degradation may have subsequently been used as controls. This could have confounded the association between the ACE I/D polymorphism and ACEI-related cough.

It is not clear why the association between the ACE I/D polymorphism and ACEI-related cough was more significant in subjects >60 y compared to those ≤60 y. It is known that older subjects have an age-related attenuation of the cough reflex; this may be related to an attenuation of bradykinin metabolism [48]. Moreover, we speculate that both the formation and the inactivation of bradykinin may decrease with age. This could lead to insufficient inactivation of bradykinin with ACEI loading in older individuals (>60 y). Thus, age-dependent differences in bradykinin metabolism may contribute to the observed age-related differences in the association between the ACE I/D polymorphism and ACEI-related cough.

Our meta-analysis may also provide some guidance in the clinical use of ACEIs. Young patients with ACEI-related cough may be advised to adhere to ACEI treatment for 2 or 3 months to observe whether the cough ceases spontaneously. In cases where the cough ceases or becomes tolerable, the patient can continue ACEI therapy. In older patients with ACEI-related cough, it would also be advisable to adhere to ACEI treatment for a set period of time. This is because ACE activity directly affects the cough reflex in older patients, and this effect was negatively associated with pneumonia risk [49], particularly in older individuals with ACE II/ID genotypes [50].

During our manuscript revision, we noted that Dr. Nishio’s group performed a similar meta-analysis [51]. Interestingly, their results were consistent with ours in the conclusion that, in Caucasians, the ACE I/D polymorphism was not associated with ACEI-related cough. However, the two studies came to different conclusions for Asians. In their meta-analyses, the ACE I/D polymorphism was associated with ACEI-related cough in Asians. In contrast, we concluded the same association, but the effect size depended on the mean age of the subjects and the follow-up duration. These results inspired us to understand the mechanism underlying ACEI-related cough. Note that the eleven studies included in our meta-analyses were not the same eleven studies included in Dr. Nishio’s meta-analyses. We identified 18 studies, but excluded 6 studies, because they were not within Hardy–Weinberg equilibrium. One other study was excluded because the frequencies of the genotypic distributions could not be extracted from the paper (for specifics, see the Results section).

Some limitations of the present study should be mentioned. First, although we excluded six studies that were not within Hardy–Weinberg equilibrium, the studies that we included had inconsistent designs. The categories and dosages of ACEIs varied among studies, and the follow-up duration ranged from 0.5 to 36 months. Second, the sample sizes were relatively small, and only five studies were age and sex matched. Thus, the results of the present meta-analysis should be interpreted with some caution. Third, some misclassification in case-control studies may have occurred. Some subjects that could tolerate a slight ACEI-related cough may have been classified as controls rather than cases.

The present study indicated that the ACE I/D polymorphism may be a genetic, age-dependent risk factor for ACEI-related cough. Large case-control studies should be performed to verify the conclusions of our meta-analysis. Future investigations aimed at elucidating the age-dependence of the association between the ACE I/D polymorphism and ACEI-related cough may help to clarify the mechanism of ACEI-related cough.

## Supporting Information

Figure S1 **PRISMA 2009 Flow Diagram.**
(TIF)

Table S1 **PRISMA Checklist.**
(DOC)
