# Supplementary figures and images for: Angiotensin-Converting Enzyme (ACE) Gene Insertion/Deletion Polymorphism and ACE Inhibitor-Related Cough: A Meta-Analysis
Source: PLoS One. 2012 Jun 19;7(6):e37396. doi: 10.1371/journal.pone.0037396 (PMC3378563; doi:10.1371/journal.pone.0037396)

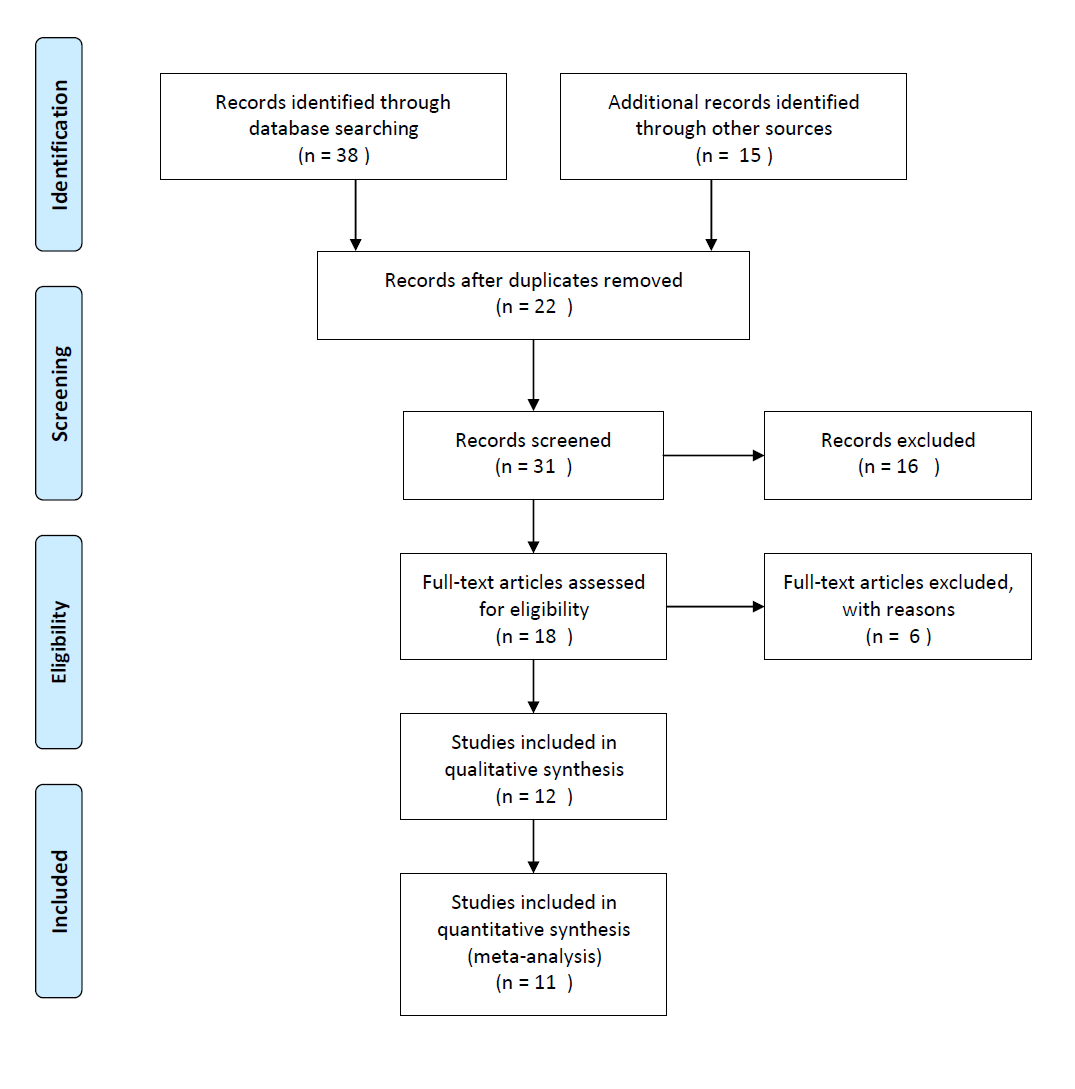

Supplement: Figure S1 — PRISMA 2009 Flow Diagram. (TIF) [file pone.0037396.s001.tif]
